# Hyperexcitable superior colliculus and fatal brainstem spreading depolarization in a model of Sudden Unexpected Death in Epilepsy

**DOI:** 10.1093/braincomms/fcac006

**Published:** 2022-01-19

**Authors:** Stuart M. Cain, Louis-Philippe Bernier, Yiming Zhang, Andrew C. Yung, Jennifer Kass, Barry Bohnet, Yi Yang, Rayshad Gopaul, Piotr Kozlowski, Brian A. MacVicar, Terrance P. Snutch

**Affiliations:** 1 Djavad Mowafaghian Centre for Brain Health, University of British Columbia, Vancouver, Canada; 2 Michael Smith Laboratories, University of British Columbia, Vancouver, Canada; 3 UBC MRI Research Centre, University of British Columbia, Vancouver, Canada

**Keywords:** SUDEP, spreading depolarization, epilepsy, Cav2.1, FHM-1

## Abstract

Cardiorespiratory arrest and death in mouse models of sudden unexpected death in epilepsy occur when spreading depolarization is triggered by cortical seizures and then propagates to the brainstem. However, the critical brain regions and the specific changes required to allow spreading depolarization to propagate to the brainstem under the relatively rare circumstances leading to a fatal seizure are unknown. We previously found that following cortical seizure-inducing electrical stimulation, spreading depolarization could occur in both the superior and inferior colliculi in Cacna1a^S218L^ mice, but was never observed in wild-type animals or following non-seizure-inducing stimuli in Cacna1a^S218L^ mice. Here, we show that optogenetic stimulation of the superior/inferior colliculi in Cacna1a^S218L^ mice induces severe seizures, and resulting spreading depolarization in the superior/inferior colliculi that propagates to the brainstem and correlates with the respiratory arrest followed by cardiac arrest. Further, we show that neurons of the superior colliculus in Cacna1a^S218L^ mice exhibit hyperexcitable properties that we propose underlie a distinct susceptibility to spreading depolarization. Our data suggest that the susceptibility of the superior colliculus to elicit fatal spreading depolarization is a result of either genetic or seizure-related alterations within the superior colliculus that may involve changes to structure, connectivity and/or excitability.

## Teaser

Cacna1a^S218L^ mice are susceptible to spontaneous, fatal seizures and premature death. Co-stimulation of the superior and inferior colliculi in Cacna1a^S218L^ mice initiates a spreading depolarization cascade that propagates to the brainstem correlating with respiratory arrest. Superior colliculus neurons display hyperexcitable action potential firing not observed in inferior colliculus neurons.

## Graphical Abstract

**Figure:**
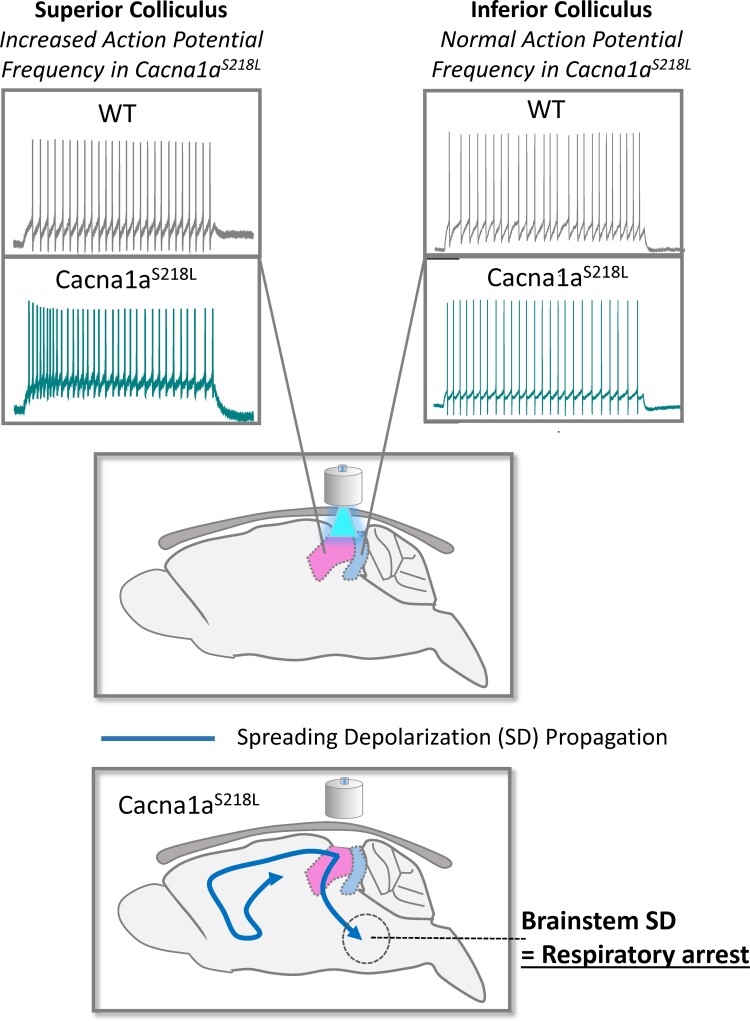

**See Gonzalez-Sulser (https://doi.org/10.1093/braincomms/fcac097) for a scientific commentary on this article.**

## Introduction

Recent studies indicate that cardiorespiratory arrest during sudden unexpected death in epilepsy (SUDEP) occurs in tandem with brainstem spreading depolarization (SD), potentially inducing suppression of brainstem cardiorespiratory centers.^1–4^ While brainstem SD is implicated in fatal cardiorespiratory arrest, it is not necessarily the focal point where SD initiates. Identifying upstream brain regions involved in the propagation of SD to the brainstem during fatal seizures is a critical step towards understanding the pathophysiology of SUDEP and to defining brain imaging biomarkers that could identify at-risk patients.

Utilizing diffusion-weighted MRI (DW-MRI) to visualize the spatiotemporal dynamics of SD, we previously reported that non-seizure-inducing cortical electrical stimulation results in subcortical SD in Familial Hemiplegic Migraine type-1 (FHM-1) knock-in mouse models. Specifically, in homozygous mice harbouring the S218L mutation in *Cacna1a* (Cacna1a^S218L^) encoding the Ca_V_2.1 (P/Q-type) calcium channel, SD propagated from the cortex into the striatum and hippocampus, whereas in wild-type mice, SD was always restricted to the cortex.^5^ Subsequently, in a study of seizure-induced SD in homozygous Cacna1a^S218L^ mice, it was possible to discriminate between spatiotemporal SD dynamics occurring during fatal versus non-fatal seizures, a critical advance towards understanding the pathophysiology underlying SUDEP.^4^ In response to seizure-inducing stimuli, wild-type mice exhibited cortical SD, but subcortical SD or fatal seizures were never observed. Conversely, in Cacna1a^S218L^ mice, while seizure-inducing stimuli induced cortical SD, propagating to the striatum and hippocampus, additionally SD occasionally propagated to the superior/inferior colliculi and thalamus. Of particular note, during fatal seizures, SD also propagated to the brainstem, correlating with respiratory arrest followed by cardiac arrest.^4^

These previous studies led us to hypothesize that during normal conditions, the thalamus and/or colliculi act as a protective gate, preventing the propagation of SD into the brainstem. Further, that under pathophysiological circumstances, such as genetic predisposition or seizure-induced structural reorganization, SD in these regions can occasionally propagate to the brainstem resulting in respiratory arrest and asphyxia.

## Materials and methods

### Animals

All experiments were performed in accordance with the guidelines of the Canadian Council on Animal Care and the University of British Columbia Animal Care Committee. Heterozygous breeding of transgenic Cacna1a^Wild-type/S218L^ mice produced male and female, wild-type and Cacna1a^S218L^ littermates for use in all experiments at post-natal day (P) 25–P40 mice and were genotyped as previously described.^6^

### Surgery

Animals (male and female) were anaesthetized using dexmedetomidine/midazolam/fentanyl anaesthesia and placed in a stereotaxic frame and an incision made in the scalp. Holes were drilled over the right hemisphere, specific to the brain region being stimulated and 0.5 µl AAV-hsyn-hChR2(H134R)-EYFP-AAV5 [AAV-hChR2; Canadian Neurophotonics Platform Viral Vector Core Facility (RRID:SCR_016477)] injected unilaterally: superior colliculus (SC)/inferior colliculus (IC) (in mm) =  bregma −4.2, midline 0.5, depth (from brain surface) 0.3; thalamus = bregma −1.7, midline 1.2, depth (from brain surface) 2.2.

Dental cement causes distortion of the DW-MRI signal; therefore, only for free-moving experiments and at the same time as AAV-hChR2 injection, fibre-optic hardware was implanted. For stimulation of the SC/IC, a port (Shanghai Laser Optical Company) was cemented (VenusFlow) onto the skull, 0.3 mm posterior to the hole over the injected area to accommodate a fibre-optic ferrule, taking care to leave a cement-free area for the laser path to the skull. For thalamic stimulation, a fibre-optic cannula (2.7 mm—the extra 0.5 mm to account for skull depth; Shanghai Laser & Optics Century) was inserted at the same depth as hCh2 injection, through the same hole used for injection and cemented (VenusFlow) onto the skull. For DW-MRI experiments, no fibre-optic hardware was implanted. The scalp incision was sutured and the animal monitored for a 2-week recovery period.

### Free-moving optogenetic stimulation of brain regions

Animals were placed in a video recording cage and an optical fibre connected to either the port (SC/IC) or cannula ferrule (thalamus). The optic fibre delivered light from a laser source (473 nm, 300 mW DPSS; Shanghai Laser & Optics Century). A 1 min baseline was acquired then stimulation applied for 2 s at 60% (6–15 mW; measured on four different fibre-optic cables utilized throughout study). If no seizure occurred, the stimulation intensity was increased in 5 min intervals: 2 s at 80% (50–110 mW), 2 s at 90% (60–150 mW), 5 s at 90% and 10 s at 90% until the stimulation threshold for a seizure was reached. Once a seizure occurred, the animal was continuously monitored.

### *Ex vivo* imaging confirmation of channel rhodopsin expression

At the end of the experiment, mice were euthanized with isoflurane, the brain removed and transferred to paraformaldehyde (4%) for 24 h before being transferred to PBS for a minimum of 2 days. Brains were then cut into slices (300 µm thickness) using a vibrating blade microtome (VT-1200S, Leica) in the coronal plane. Imaging was performed using a Zeiss AxioZoomV16 fluorescence stereo microscope with a Plan-NEOFLUAR Z 2.3×/1.5 NA objective using a GFP/YFP filter set (FS38: excitation 470/40, emission 525/50).

### Magnetic resonance imaging

Animals previously injected with AAV-hChR2 were anaesthetized using isoflurane and an incision was made in the scalp. For stimulation of the SC/IC, a port (Shanghai Laser & Optics Century) was glued onto the skull, 0.3 mm posterior to the hole over the injected area to accommodate a fibre-optic ferrule, taking care to leave a cement-free area for the laser path to the skull. For thalamic stimulation, a fibre-optic cannula (2.7 mm, the extra 0.5 mm to account for skull depth; Shanghai Laser & Optics Century) was inserted at the same depth as hCh2 injection, through the hole used for injection and glued onto the skull. To transfer onto an injectable anaesthetic, animals were then injected with urethane (30%, 8 µl/g mouse weight) and isoflurane gradually decreased to 0% over 2 min. A minimum period of 20 min was applied between cessation of isoflurane and initiation of scanning. During scanning, animals breathed unaided on normal air delivered to the scanner by a facemask.

DW-MRI was undertaken using a 7 T animal scanner (Bruker Biospin Ltd). A quadrature radiofrequency (RF) coil with 70 mm inner diameter volume was used for pulse transmission and the MRI signal was received with a 14 mm diameter actively decoupled surface coil. The mouse was laid supine in the MRI cradle with the fibre-optic cable fed through the centre of the RF coil and connected to the port or cannula ferrule. Respiratory rate, heart rate and body temperature were monitored during scanning with a Model 1025 Control/Gating system (SA Instruments). DW-MRI was acquired using DW spin-echo echo planar imaging with a *b*-value of 1800 s/mm^2^ (echo time/repetition time = 29/2000 ms, two shots, field of view = 2 × 2 cm, matrix size = 64 × 64, slice thickness = 1.25 mm, eight interleaved slices). Of note, in a refinement from our previous studies, modifications to the custom RF coil, combined with the software upgrades (Bruker Paravision) and better magnetic field shimming allowed the use of two shots instead of four shots, resulting in a 4 s time resolution compared with 8 s used previously, without impairment of image resolution.

Upon initiation of scanning, a 1 min baseline was acquired followed by stimulation for 2 s at 60% (6–15 mW; measured on four different fibre-optic cables utilized throughout study). If no seizure occurred, the stimulation intensity was increased in 5 min intervals: 2 s at 80% (50–110 mW), 2 s at 90% (60–150 mW), 5 s at 90% and 10 s at 90% until the stimulation threshold for SD was reached.

### Acute brain slice electrophysiology

Mice were briefly anaesthetized using isoflurane, killed by cervical dislocation, the brains rapidly removed and transferred to ice-cold sucrose solution containing in mM: 234 sucrose, 24 NaHCO_3_, 1.25 NaH_2_PO_4_, 11 glucose, 2.5 KCl, 0.5 CaCl_2_, 6 MgCl_2_, bubbled with 95% O_2_:5% CO_2_. Brain tissue was glued to a cutting chamber (VT-1200, Leica), which was then filled with ice-cold sucrose solution. Brain slices (350 μm thick) were cut in the coronal plane from bregma −5.3 to −4.00 mm (SC/IC). Brain slices were incubated at 33–35°C in artificial CSF (aCSF) for a minimum of 1 h at 34°C in aCSF containing in mM: 126 NaCl, 2.5 KCl, 26 NaHCO_3_, 1.25 NaH_2_PO_4_, 2 CaCl_2_, 2 MgCl_2_, 10 glucose; bubbled with 95% O_2_:5% CO_2_.

Slices were transferred to a recording chamber superfused with aCSF and maintained at 33–35°C. Neurons were visualized using a DIC microscope and infrared camera (Slicescope 6000, Scientifica, UK) and visually identified by their location, morphology and orientation. All recordings were undertaken using a Multiclamp 700B amplifier and pClamp software version 10 (Molecular devices). The recording chamber was grounded with an Ag/AgCl pellet.

Whole-cell current-clamp recordings were undertaken using fire polished borosilicate glass pipettes (4–6 MΩ) filled with the intracellular solution containing in mM: 120 K-gluconate, 10 HEPES, 1 MgCl_2_, 1 CaCl_2_, 11 KCl, 11 EGTA, 4 MgATP, 0.5 NaGTP, pH adjusted to 7.2 using KOH and osmolarity adjusted to 290 mOsm/kg using d-mannitol. The liquid junction potential for current-clamp solutions was calculated as +13.3 mV and corrected offline. To evaluate basic input–out action potential frequency response to hyperpolarization and depolarization, DC current was injected from −110 to +200 pA in 10 pA increments for a duration of 1000 ms at the cell’s intrinsic resting membrane potential. Membrane potential responses under current-clamp conditions were sampled at 50 kHz and filtered at 10 kHz. Bridge balance was monitored during recordings and any neurons displaying bridge balance values >20 MΩ were discarded. Capacitance neutralization was performed between 3.8 and 4.2 pF.

Whole-cell voltage-clamp recordings were performed using the same intracellular solution and external aCSF solution with the exception that to record sEPSCs picrotoxin (100 µM) was added to the aCSF. To evaluate sEPSCs, cells were held at a membrane potential of −50 mV during a 60 s gap-free recording. Data acquisition was sampled at 20 kHz and filtered at 2.4 kHz. Recordings with a series resistance of >25 MΩ were discarded and series resistance was compensated to 70%.

### mRNA analysis using quantitative real-time PCR

For calcium channel subunit analysis, SC and IC tissue was dissected from 500 µm horizontal brain slices acquired from three individual animals from each genotype followed by total RNA extraction.

Each sample was homogenized in the presence of TRI-Reagent (Ambion, AM9738) and the total RNA was isolated using a MagMAX^™^-96 for Microarrays preparation kit (Invitrogen, AM1839). Total cDNA was then synthesized from total RNA using a High Capacity cDNA Reverse Transcription kit (Applied Biosystems, 4368814). A 2720 Thermal Cycler (Applied Biosystems) was used to perform cDNA synthesis and the procedure was 25°C for 10 min, 37°C for 120 min and 85°C for 5 s. Gene-specific qPCR reactions were performed in triplicate using the KAPA Probe Fast qPCR Master Mix (2×) (KAPA Biosystems, KM4702). The qPCR probes for the Ca_V_ α_1_ subunits were synthesized by ThermoFisher Scientific. Quantitative PCR reactions were performed on a C1000 Touch Thermal Cycler Touch TM Real-Time (PCR Detection) System (Bio-Rad), using cycling conditions of 95°C for 3 min (95°C for 15 s, 60°C for 45 s) ×40 cycles.

The probe efficiency was measured by titrating isoform-specific probes against a cDNA sample. Copy numbers for each isoform in each sample were then calculated and scaled, relative to GAPDH. Target and control probe reactions were run in triplicate and averaged for each sample.

### Data and statistical analysis

Electrophysiological data analysis was performed using the Clampfit (v10, Molecular Devices). DW-MRI analyses were performed using the MATLAB (v 2014a, Mathworks) and ImageJ (v 1.50d, NIH). Graphing and statistical analyses were performed using the Origin (v8.6, OriginLab). Data followed a normal distribution and a statistical significance was calculated using Student’s two-sample *t*-test (paired where relevant). One-way ANOVA with Tukey’s *post hoc* test was used for multiple comparisons. Data are plotted as mean ± standard error.

### Data availability

Raw data were generated at the University of British Columbia. Derived data supporting the findings of this study are available from the corresponding author on request.

## Results

### Stimulation of the SC/IC induces fatal seizures in Cacna1a^S218L^ mice

**Figure 1:**
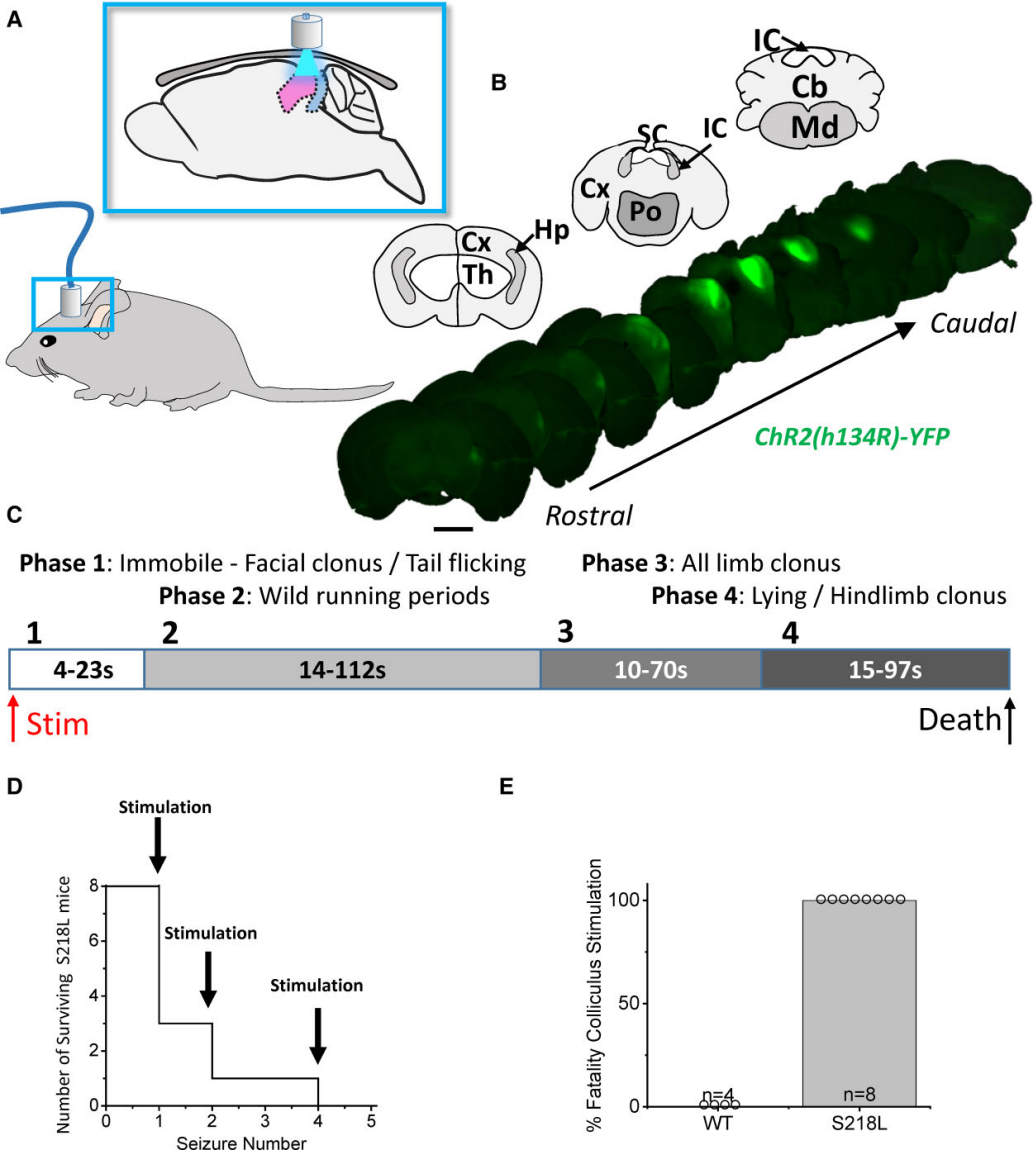
**Stimulation of the colliculi induces fatal seizures in Cacna1a^S218L^ mice.** (**A**) Schematic showing the location of fibre-optic port on the head (lower panel) and through-skull optogenetic stimulation relative to the superior and inferior colliculus in the sagittal view (upper panel). Scattering of light through the skull and AAV spread results in stimulation of both regions. (**B**) Upper panels show coronal maps corresponding to coronal *ex vivo* immunofluorescence images of AAV5-hChR2(H134R)-YFP expression in lower panels, arranged from rostral (bregma = −3.0 mm) to caudal (bregma = −6.0 mm). Note that lateral cortical lobes are lost during sectioning in some sections. (**C**) Upper schematic displays the timing of seizures in Cacna1a^S218L^ mice induced by stimulation of colliculi defined by four distinct behaviours. (**D**) Line graph shows fatality occurring relative to the number of stimulation-induced seizures was induced in individual Cacna1a^S218L^ mice. Stimulations on surviving mice were separated by 30 min. (**E**) Bar chart shows the number of Cacna1a^S218L^ mice (*n* = 8; *m* = 5, *f* = 3; no sex-related difference) displaying fatal seizures relative to lack of fatality (or seizures) in wild-type mice (*n* = 4; *m* = 2, *f* = 2; no sex-related difference). Scale bars, 2 mm.

To establish whether the SD initiated in the SC/IC in isolation can propagate to the brainstem and induce respiratory arrest, under anaesthesia, the SC of wild-type and Cacna1a^S218L^ mice were stereotaxically injected with AAV.hChR2(H134R).YFP and a fibre-optic port was fitted on the intact skull next to the injection site. Following a 3-week recovery, the superior and inferior colliculi were stimulated remotely through the skull using a 473 nm laser light source coupled to an optical fibre inserted into the skull port while freely moving (Fig. 1A). Channelrhodopsin [ChR2; hChR2(H134R)] expression was verified post-mortem noting that AAV spread to the IC inducing ChR2 expression in both SC/IC (Fig. 1B). While optogenetic stimulation (1–10 s continuous pulse) of the SC/IC in wild-type mice did not lead to any seizure or fatality events (Fig. 1E), the same stimulation in Cacna1a^S218L^ mice induced severe seizures, characterized by stereotyped behaviour initiating as facial clonus, followed by wild running, all-limb clonus and terminating with hindlimb clonus (Fig. 1C). In five out of eight Cacna1a^S218L^ mice, respiratory arrest and death occurred during hindlimb clonus on the first seizure event (Fig. 1D). Following recovery, the three surviving mice died of respiratory arrest during hindlimb clonus during subsequent stimulation of the SC/IC (Fig. 1E).

### Stimulation of the SC/IC initiates SD that propagates to the brainstem arresting respiration in Cacna1a^S218L^ mice

**Figure 2:**
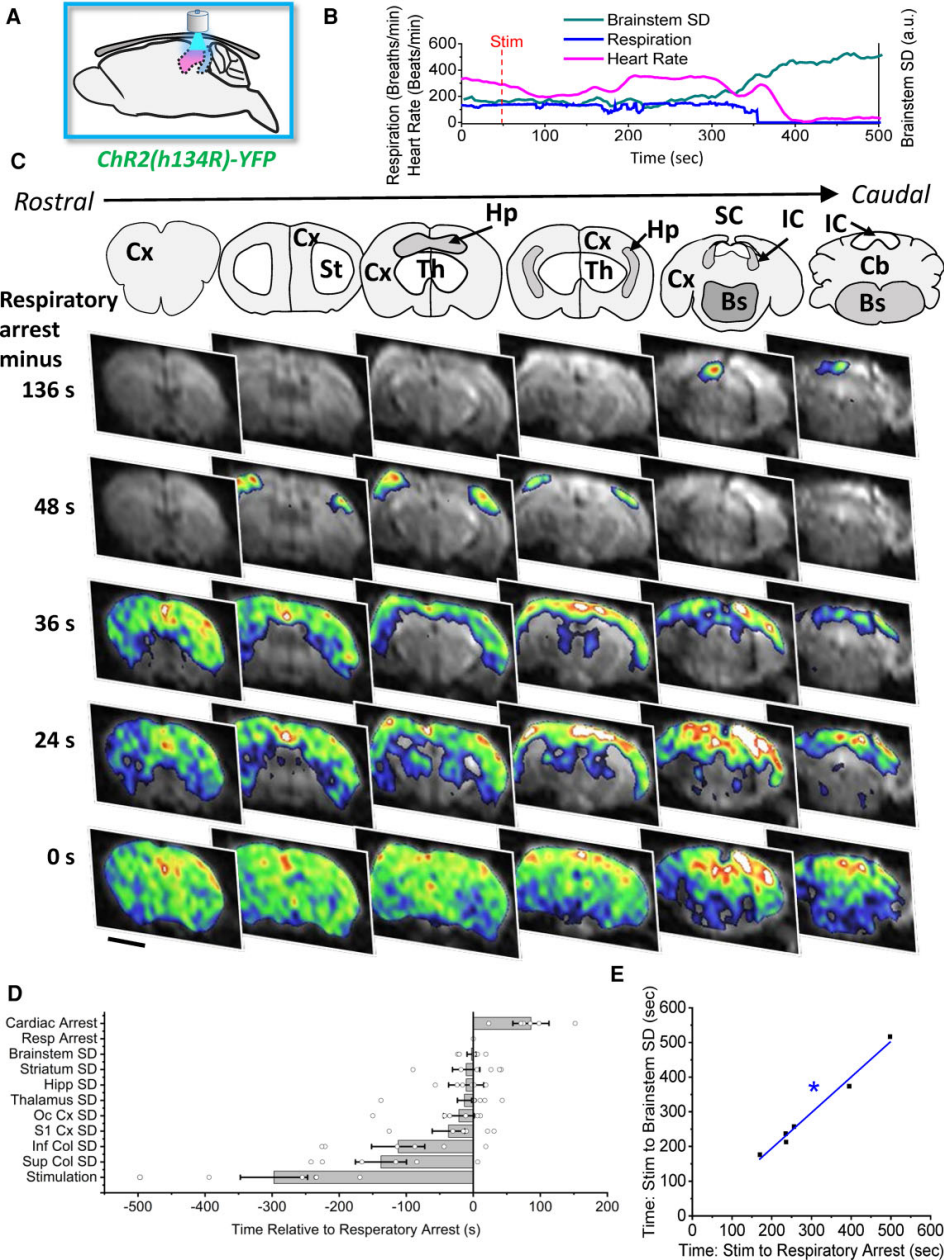
**Stimulation of SC/IC induces SD that propagates to brainstem inducing respiratory arrest in Cacna1a^S218L^ mice**. (**A**) Schematic demonstrating SC/IC optogenetic stimulation for 2 s through-skull initiating SD in the SC/IC in Cacna1a^S218L^ mice (*n* = 6, *m* = 4, *f* = 2; no sex-related difference). SD was not observed in wild-type mice (*n* = 6, *m* = 3, *f* = 3; no sex-related difference). (**B**) Representative SD, respiratory and heart rate traces from a single Cacna1a^S218L^ animal showing that brainstem SD occurs simultaneously with respiratory arrest, followed by cardiac arrest (a.u., arbitrary units). (**C**) Upper panels show coronal maps corresponding to representative DW-MRI data in lower panels showing coronal slices of the brain at sequential levels from rostral (left) to caudal (right) and at different time points relative to respiratory arrest (top to bottom). SD initiated in the SC/IC, propagated to the cortex, then through subcortical structures and lastly into the brainstem. Cx, cortex; St, striatum; Hp, hippocampus; Th, thalamus; SC, superior colliculus; IC, inferior colliculus; Bs, brainstem; Cb, cerebellum. (**D**) SD initiation during fatal seizures in each subcortical brain structure relative to the onset of respiratory arrest. (**E**) Initiation of brainstem SD demonstrated strong correlation with respiratory arrest in fatal seizures as measured by time from stimulation. Scale bars, 2 mm. *Adjusted *R*^2^ value = 0.98.

To establish whether stimulation of the SC/IC induces SD, we performed optogenetic stimulation of the SC/IC using the same parameters, except that the animal was anaesthetized and imaged with DW-MRI scanning with continuous respiratory and cardiac monitors. DW-MRI shows an increase in image intensity in areas of brain swelling, which occurs concurrently with SD, across the entire brain (in eight coronal slices) with a 4 s time resolution.^4,5,7,8^ While stimulation of the SC/IC did not induce SD, DW-MRI movement artefacts or fatalities in wild-type mice, in six out of eight Cacna1a^S218L^ mice optogenetic stimulation-initiated SD in the SC/IC, which then propagated to the cortex, travelling around the cortical hemisphere from the dorsal–caudal to ventral–rostral cortex (Fig. 2). In addition, SD propagated back into the SC/IC along with the hippocampus, thalamus and striatum, then finally travelling in a dorsal to ventral direction into the brainstem. Respiratory arrest occurred in all six Cacna1a^S218L^ mice where SD was initiated in the SC/IC, correlating strongly with initiation of SD in the brainstem (*R* = 0.98; Fig. 2E). DW-MRI movement artefacts were observed throughout SD propagation, indicative of seizures. In the remaining two Cacna1a^S218L^ mice where no SD occurred following SC/IC opto-stimulation, no seizures or fatality were observed.

### Stimulation of the dorsal thalamus initiates SD propagation without respiratory arrest

**Figure 3:**
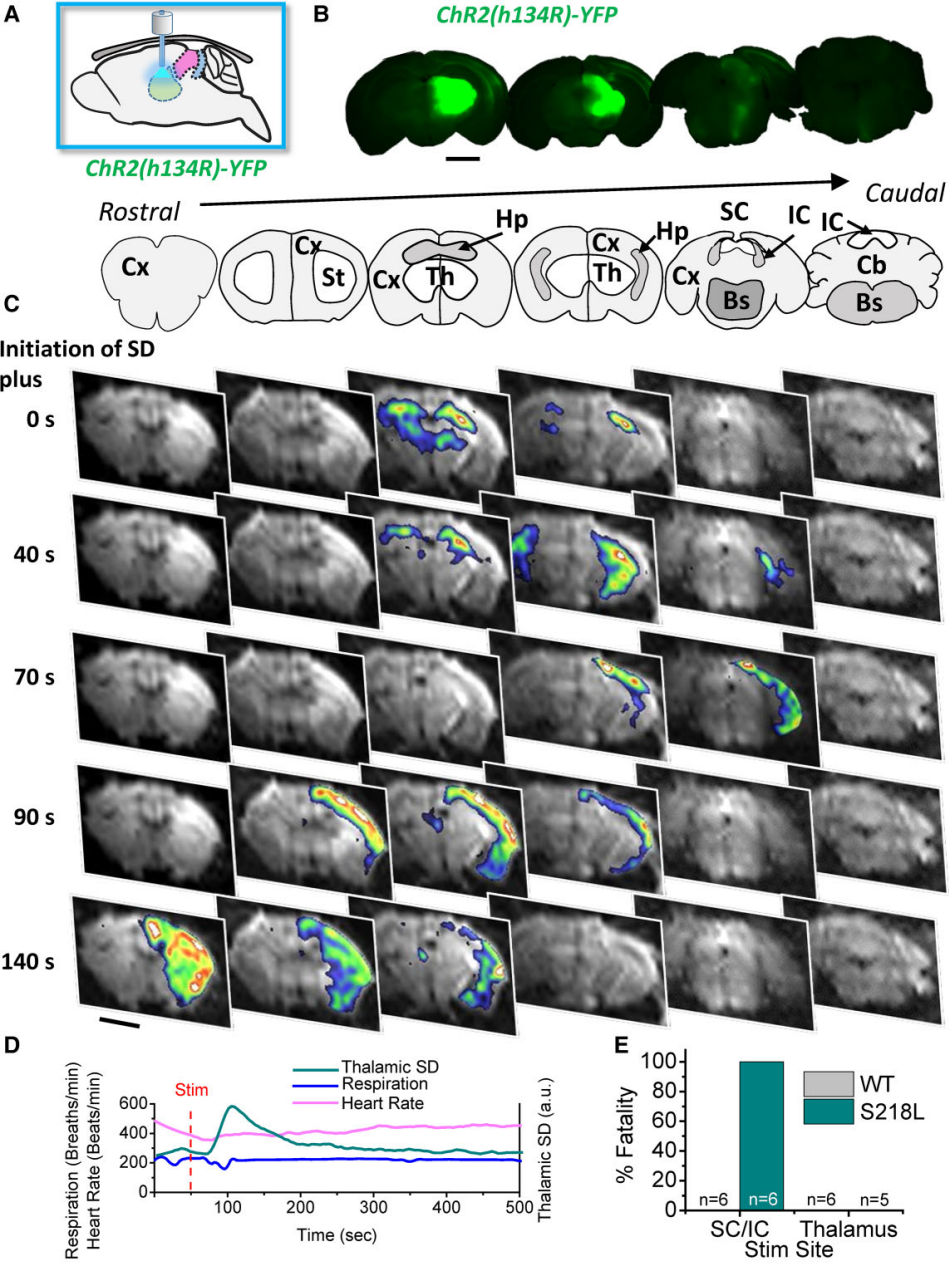
**Stimulation of the thalamus induces non-fatal SD that is restricted to the cortex/hippocampus and spares the brainstem in Cacna1a^S218L^ mice.** (**A**) Schematic demonstrating thalamic optogenetic stimulation for 2 s via a fibre-optic cannula implanted directly into the dorsal thalamus. (**B**) Upper panels show coronal *ex vivo* immunofluorescence images of AAV5-hChR2(H134R)-YFP expression corresponding to coronal maps in (**C**; upper panels). (**C**) Upper panels show coronal maps corresponding to representative DW-MRI data in lower panels showing coronal slices of the brain at sequential levels from rostral (left) to caudal (right) and at different time points relative to respiratory arrest (top to bottom). Optogenetic stimulation-initiated SD in the dorsal thalamus, which propagated to the hippocampus then throughout the cortex. (**D**) Representative SD, respiratory and heart rate traces from a single Cacna1a^S218L^ animal showing that thalamic SD does not induce respiratory or cardiac arrest (a.u., arbitrary units). (**E**) Bar chart summarizing % fatality observed with SC/IC versus thalamic stimulation in Cacna1a^S218L^ mice (S218L; Thalamic stimulation; *n* = 6; m = 4, f = 2; no sex-related difference) and wild-type (WT; Thalamic stimulation; *n* = 5; m = 3, f = 2; no sex-related difference) mice. Scale bars, 2 mm.

In our previous study, in addition to SD occurring in the SC/IC during seizure events in Cacna1a^S218L^ mice, we also occasionally observed thalamic SD. In order to determine whether fatal SD is specific to stimulation of the SC/IC, ChR2 was injected into the dorsal thalamus and an optic cannula inserted at the injection site. Following a 3-week recovery, DW-MRI scanning was performed simultaneously with optogenetic thalamic stimulation under anaesthesia (Fig. 3A and B). During the cortical and hippocampal SD propagation DW-MRI, movement artefacts were occasionally observed indicative of seizures, but no fatalities were observed in either Cacna1a^S218L^ or wild-type mice (Fig. 3E) and there were no effects observed on respiration or heart rate. In Cacna1a^S218L^ mice, SD initiated in the dorsal thalamus and rapidly propagated to the hippocampus, then throughout the cortex (Fig. 3C and D). SD did not invade any other brain structures, including the SC/IC or brainstem. Thalamic SD could not be induced in wild-type animals; however, thalamic opto-stimulation occasionally induced hippocampal SD alone. We speculate this effect in wild-type mice was either due to a combination of the spread of AAV-ChR2 expression into the hippocampus and light reflected back from brain tissue ventral to the end of the optic cannula or via synaptic activation by stimulation of thalamo-hippocampal collaterals.

### The SC in Cacna1a^S218L^ mice is selectively hyperexcitable

**Figure 4:**
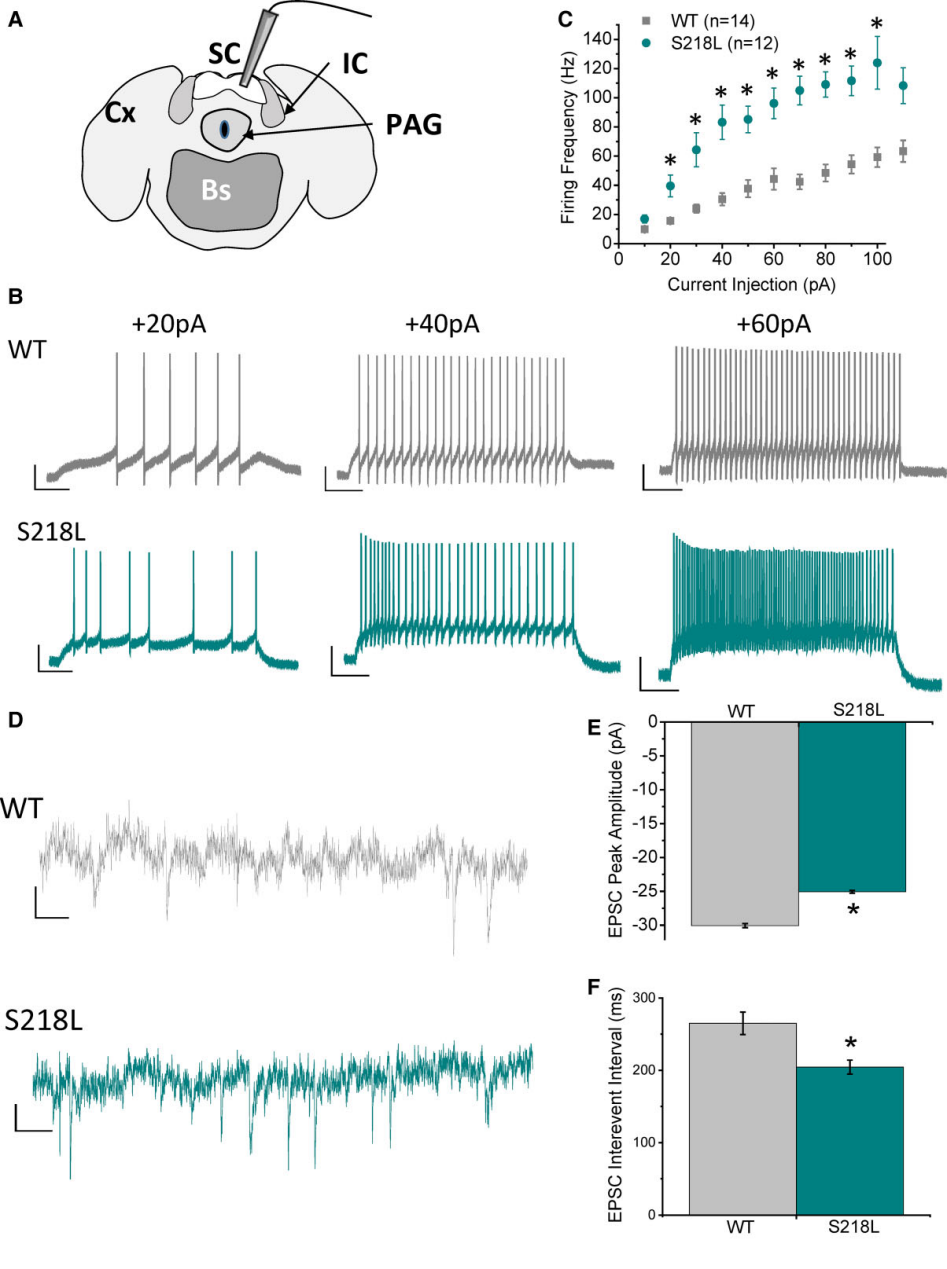
**SC neurons in Cacna1a^S218L^ mice display hyperexcitable intrinsic firing and synaptic excitability.** (**A**) Schematic of acute brain slice cut in the coronal plane for whole-cell patch-clamp recording of SC (granule layer) neurons. PAG, periaqueductal grey. (**B**) Representative current-clamp traces recorded in SC neurons from wild-type and Cacna1a^S218L^ mice, demonstrating tonic firing response to increasing current injections (scale bars, 200 ms, 20 mV) (WT *n* = 15; *m* = 7, *f* = 8; S218L *n* = 11; *m* = 6, *f* = 5; no sex-related difference). (**C**) Mean input–output response data of tonic action potential frequency in response to increasing current injection over threshold. (**D**) Representative voltage-clamp traces recorded in wild-type and Cacna1a^S218L^ mice (scale bars,  20 ms, 10 pA), demonstrating (**E**) decreased amplitude and **(F)** decreased inter-event interval (increased frequency) of spontaneous glutamatergic synaptic activity (sEPCSs). *P < 0.05 two-sample *t*-test.

To examine the apparent susceptibility of the SC/IC to SD whole-cell patch-clamp analysis of neuronal electrophysiological properties was undertaken using acute brain slices (Fig. 4A). Input (injected current) to output (tonic action potential firing frequency) response was significantly increased in Cacna1a^S218L^ SC neurons (Fig. 4B and C), which yielded a significantly larger slope constant compared with wild-type when individually fitted with a linear curve (wild-type = 0.8 ± 0.16, Cacna1a^S218L^ = 1.43 ± 0.17; *P* = 0.02). Wild-type and Cacna1a^S218L^ SC neurons displayed no significant differences in either resting membrane potential (wild-type = −70.8 ± 2.1 mV, Cacna1a^S218L^ = −73.3 ± 1.1 mV; *P* = 0.28) or input resistance (wild-type = 494 ± 74 MOhm, Cacna1a^S218L^ = 564 ± 67 MOhm; *P* = 0.50). Spontaneous glutamatergic synaptic activity was also assessed, which displayed significantly smaller amplitude but higher frequency (decreased inter-event interval) of sEPSCs in Cacna1a^S218L^ SC neurons compared with wild-type SC neurons (Fig. 4D–F). Conversely, IC neurons displayed no significant differences in input–output responses (Supplementary Fig. 1A–C) or spontaneous glutamatergic activity (Supplementary Fig. 1D–F) in Cacna1a^S218L^ SC neurons compared with wild-type SC neurons indicating the selective hyperexcitability of the SC over the IC.

FHM-1 results from gain-of-function missense mutations in the Ca_V_2.1 (P/Q-type) voltage-gated calcium channel, encoded by the *CACNA1A* gene. To test for gross changes in calcium channel expression in the SC and IC, we examined mRNA transcript levels of all 10 calcium channel α_1_-subunit genes between wild-type and Cacna1a^S218L^ mice. There were no detectable changes in expression between Cacna1a^S218L^ and wild-type mice in P/Q-type channel or other high-threshold calcium channel in either the SC or IC (Supplementary Fig. 2). We did, however, observe decreased transcript levels of the Ca_V_3.1 and Ca_V_3.3 low-threshold T-type calcium channels in the SC of Cacna1a^S218L^ mice (Supplementary Fig. 2). Of note, alterations in T-type calcium channel expression/activity are observed in a number of animal epilepsy models.^9,10^

## Discussion

We and others previously reported that SD invades distinct subcortical structures during non-seizure-inducing cortical stimulation (striatum, hippocampus)^5,8^ and during non-fatal seizures (striatum, hippocampus, thalamus, colliculi) in the Cacna1a^S218L^ mouse model of FHM-1 and SUDEP.^4^ Only in fatal seizures did we observe brainstem SD, occurring in conjunction with respiratory arrest and followed by cardiac arrest. In the current study, direct stimulation of the SC/IC in awake, free-moving Cacna1a^S218L^ mice resulted in severe behavioural seizures characterized by stereotyped activity lasting several minutes and terminating with respiratory arrest and fatality in ∼60% of animals upon the initial stimulation, with remaining animals dying on a subsequent stimulation. This strongly implicates the SC/IC as a critical node of the seizure-induction network in Cacna1a^S218L^ mice. The SC and IC have been previously implicated as a potential seizure foci in the Genetic Epilepsy Prone Rats-9 (GEPR-9) absence epilepsy model,^11^ which display audiogenic seizures and an associated increase in high-voltage threshold calcium channel expression.^12,13^ Whole-cell patch-clamp was utilized here to provide insight into the potential mechanisms underlying hyperexcitability in the SC/IC and whether one or both regions were intrinsically excitable in Cacna1a^S218L^ mice. It is apparent that the SC alone is selectively hyperexcitable, exhibiting higher output frequency action potential firing in response to increased input depolarization and higher frequency spontaneous glutamatergic synaptic activity in Cacna1a^S218L^ SC neurons compared with the wild-type SC neurons. We speculate that higher frequency tonic firing would increase the susceptibility of the SC towards depolarization and higher frequency synaptic activity would enhance afferent drive onto SC neurons. Further experiments are required to selectively stimulate the SC and IC, and to identify the specific ionic conductances underlying SC intrinsic hyperexcitability. Since the Cacna1a^S218L^ gain-of-function mutation generates a tonic inward calcium conductance at rest,^14,15^ the underlying P/Q-type channel is a likely candidate to underlie one or both hyperexcitable properties.

In the present study, the incidence of fatality in response to SC/IC stimulation was higher (five out of eight animals died on the first stimulation) than that observed in our previous study in response to cortical stimulation (4 fatal seizures, with 15 non-fatal seizures), indicating that direct stimulation of the SC/IC is more likely to induce fatality.^4^ We speculate that only a subset of cortical stimulation-induced or spontaneous seizures propagate to the SC/IC, whereas direct stimulation of the SC/IC ensures SD in this region. As a result, stimulation of SC/IC is more likely to induce fatality, but can still fail to propagate to the brainstem (three animals required a second stimulation to observe fatality). In a recent study, seizure-induced SD was proposed to occur as an innate, protective mechanism to silence brain activity during seizure events, thereby terminating epileptiform activity.^16^ If this is correct, it follows that only during aberrant pathophysiological conditions would SD propagate to the brainstem where the effect on respiratory function would be fatal. Of note, stimulation of the SC/IC induced SD that initiated in the SC/IC and then propagated through the cortex and back into the SC/IC before invading the brainstem. This raises the possibility that fatal brainstem SD initiated in the SC/IC may additionally involve altered activity in other brain regions that do not exhibit SD. However, since thalamic stimulation also induced widespread cortical and subcortical SD but without fatal brainstem SD, it follows that the SC/IC plays a specific and critical role in the propagation of fatal brainstem SD.

Seizure activity is capable of inducing the extreme depolarization required to initiate the cascade wave of SD.^3,17^ It follows that seizure activity spreading into the SC/IC likely induces SD, similar to that caused by optogenetic stimulation in the current study. The SC and IC are involved in the processing and relay of visual and auditory sensory information, respectively, and are necessary for source orientation localization.^18,19^ Efferents from the SC project to the cortex,^20^ whereas IC effects project primarily to the thalamus.^21^ In the current study, opto-stimulation of the SC/IC resulted in SD that initiated in the SC/IC and then propagated through the cortex, followed by thalamus, back through the SC/IC and finally into the brainstem. Of note, the SC also sends projections to brainstem gaze centres,^20^ although we observed a time delay of between 1 and 2 min between initiation of SD in the SC and initiation in the brainstem, which is more in line with diffusional rather than synaptic propagation of SD. Nevertheless, the appearance of SD in the brainstem correlated with respiratory arrest, which was then followed by cardiac arrest, providing convincing support to the theory that during fatal seizure events SD invades the brainstem, suppressing critical respiratory network activity and causing death by asphyxia.

Of note, in free-moving experiments, ∼40% of mice recovered from the initial hindlimb clonus and gasping that characteristically preceded death. This raises two possibilities; the first being that SD occurring in the SC/IC fails to propagate to the brainstem in non-fatal seizures and second being that brainstem SD can be reversible, both of which are findings that were observed in our previous study.^4^ These findings may have important implications for SUDEP, the obvious one being that mechanical respiration during severe seizures may prevent fatality in epilepsy patients at risk of SUDEP, if maintained for several minutes to allow reversal of brainstem SD.

In summary, Cacna1a^S218L^ mice display fatal seizures in response to direct opto-stimulation of the SC/IC by inducing SD that propagates to the brainstem and recapitulates fatality observed with spontaneous and cortically evoked seizures, but with a particularly high incidence of death. Of particular note, the SC of Cacna1a^S218L^ mice exhibit an intrinsic hyperexcitable profile that likely underlies its susceptibility to SD. These findings warrant further investigation into the SC as a key propagator of fatal brainstem SD during severe seizures and have strong relevance in the pathogenic mechanisms underlying SUDEP.

## Supplementary Material

fcac006_Supplementary_Data

## Abbreviations

Ca_V_ =: voltage-gated calcium channel
ChR2 =: channelrhodopsin
DW-MRI =: diffusion-weighted MRI
FHM-1 =: familial hemiplegic migraine type-1
IC =: inferior colliculus
SC =: superior colliculus
SD =: spreading depolarization
SUDEP =: sudden unexpected death in epilepsy

## References

[1] Aiba I , Noebels JL. Spreading depolarization in the brainstem mediates sudden cardiorespiratory arrest in mouse SUDEP models. Sci Transl Med. 2015;7(282):282ra46. 10.1126/scitranslmed.aaa4050 PMC4852131 25855492

[2] Lhatoo S , Noebels J, Whittemore V, The NINDS Center for SUDEP Research. Sudden unexpected death in epilepsy: Identifying risk and preventing mortality. Epilepsia. 2015;56(11):1700–1706. 26494436 10.1111/epi.13134 PMC4852129

[3] Devinsky O , Hesdorffer DC, Thurman DJ, Lhatoo S, Richerson G. Sudden unexpected death in epilepsy: Epidemiology, mechanisms, and prevention. Lancet Neurol. 2016;15(10):1075–1088. 27571159 10.1016/S1474-4422(16)30158-2

[4] Loonen ICM , Jansen NA, Cain SM, et al Brainstem spreading depolarization and cortical dynamics during fatal seizures in Cacna1a S218L mice. Brain. 2019;142(2):412–425. 30649209 10.1093/brain/awy325 PMC6351775

[5] Cain SM , Bohnet B, LeDue J, et al In vivo imaging reveals that pregabalin inhibits cortical spreading depression and propagation to subcortical brain structures. Proc Natl Acad Sci USA. 2017;114(9):2401–2406. 28223480 10.1073/pnas.1614447114 PMC5338525

[6] van den Maagdenberg AMJM , Pizzorusso T, Kaja S, et al High cortical spreading depression susceptibility and migraine-associated symptoms in Ca(v)2.1 S218L mice. Ann Neurol. 2010;67(1):85–98. 20186955 10.1002/ana.21815

[7] de Crespigny A , Röther J, van Bruggen N, Beaulieu C, Moseley ME. Magnetic resonance imaging assessment of cerebral hemodynamics during spreading depression in rats. J Cereb Blood Flow Metab. 1998;18(9):1008–1017. 9740104 10.1097/00004647-199809000-00010

[8] Eikermann-Haerter K , Yuzawa I, Qin T, et al Enhanced subcortical spreading depression in familial hemiplegic migraine type 1 mutant mice. J Neurosci. 2011;31(15):5755–5763. 21490217 10.1523/JNEUROSCI.5346-10.2011 PMC3135337

[9] Cain SM , Snutch TP. Voltage-gated calcium channels in epilepsy. In: Noebels JL, Avoli M, Rogawski MA, Olsen RW, Delgado-Escueta A, eds. Jasper’s basic mechanisms of the epilepsies. 4th edn. Oxford University Press; 2012:66–84.

[10] Cain SM , Tyson JR, Choi H-B, et al CaV 3.2 drives sustained burst-firing, which is critical for absence seizure propagation in reticular thalamic neurons. Epilepsia. 2018;59(4):778–791. 29468672 10.1111/epi.14018 PMC5900875

[11] Faingold CL . Neuronal networks in the genetically epilepsy-prone rat. Adv Neurol. 1999;79:311–321. 10514823

[12] N’Gouemo P , Faingold CL, Morad M. Calcium channel dysfunction in inferior colliculus neurons of the genetically epilepsy-prone rat. Neuropharmacology. 2009;56(3):665–675. 19084544 10.1016/j.neuropharm.2008.11.005 PMC2638996

[13] N’Gouemo P , Yasuda R, Faingold CL. Seizure susceptibility is associated with altered protein expression of voltage-gated calcium channel subunits in inferior colliculus neurons of the genetically epilepsy-prone rat. Brain Res. 2010;1308:153–157. 19836362 10.1016/j.brainres.2009.10.019 PMC2793592

[14] Adams PJ , Rungta RL, Garcia E, van den Maagdenberg AMJM, Macvicar BA, Snutch TP. Contribution of calcium-dependent facilitation to synaptic plasticity revealed by migraine mutations in the P/Q-type calcium channel. Proc Natl Acad Sci USA. 2010;107(43):18694–18699. 20937883 10.1073/pnas.1009500107 PMC2972937

[15] Di Guilmi MN , Wang T, Inchauspe CG, et al Synaptic gain-of-function effects of mutant Cav2.1 channels in a mouse model of familial hemiplegic migraine are due to increased basal [Ca2+]i. J Neurosci. 2014;34(21):7047–7058. 24849341 10.1523/JNEUROSCI.2526-13.2014 PMC4028489

[16] Tamim I , Chung DY, de Morais AL, et al Spreading depression as an innate antiseizure mechanism. Nat Commun. 2021;12(1):2206. 33850125 10.1038/s41467-021-22464-x PMC8044138

[17] Kramer DR , Fujii T, Ohiorhenuan I, Liu CY. Interplay between cortical spreading depolarization and seizures. Stereotact Funct Neurosurg. 2017;95(1):1–5. 10.1159/000452841 28088802

[18] Davis KA , Ramachandran R, May BJ. Auditory processing of spectral cues for sound localization in the inferior colliculus. J Assoc Res Otolaryngol. 2003;4(2):148–163. 12943370 10.1007/s10162-002-2002-5 PMC3202719

[19] Hafed ZM . Superior colliculus: A vision for orienting. Curr Biol. 2018;28(18):R1111–R1113. 30253154 10.1016/j.cub.2018.07.047

[20] May PJ . The mammalian superior colliculus: Laminar structure and connections. Prog Brain Res. 2006;151:321–378. 16221594 10.1016/S0079-6123(05)51011-2

[21] Ito T , Oliver DL. The basic circuit of the IC: Tectothalamic neurons with different patterns of synaptic organization send different messages to the thalamus. Front Neural Circuits. 2012;6:48. 22855671 10.3389/fncir.2012.00048 PMC3405314

