# Supplementary material for: Hyperexcitable superior colliculus and fatal brainstem spreading depolarization in a model of Sudden Unexpected Death in Epilepsy
Source: Brain Commun. 2022 Jan 19;4(2):fcac006. doi: 10.1093/braincomms/fcac006 (PMC9035526; doi:10.1093/braincomms/fcac006)
Supplement: fcac006_Supplementary_Data [file fcac006_supplementary_data.docx]

**Hyperexcitable Superior Colliculus and Fatal Brainstem Spreading Depolarization in a Model of SUDEP**

**
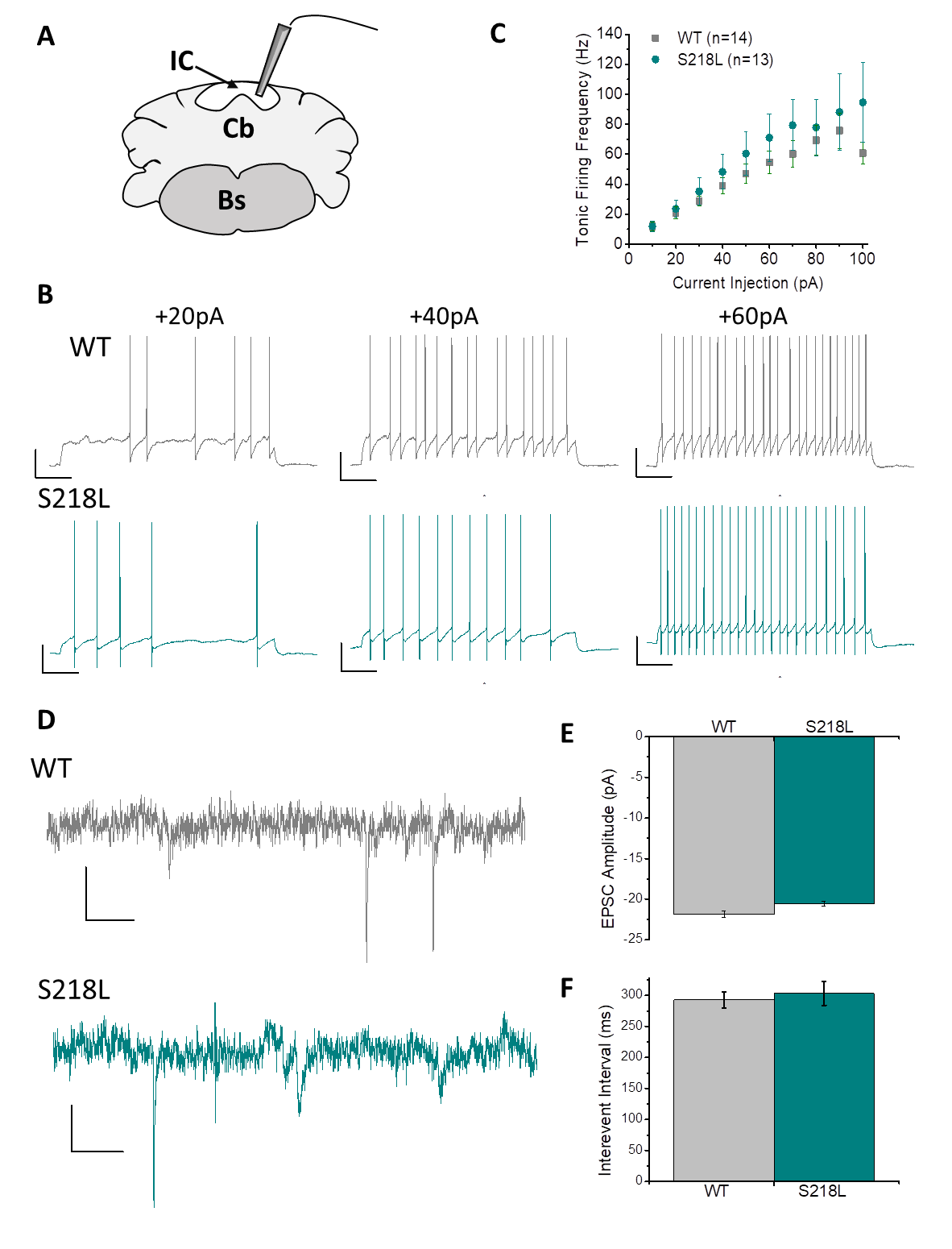
Supplementary Figure 1. IC neurons in Cacna1a^S218L^ mice do not display a hyperexcitable phenotype (a)** Schematic of acute brain slice cut in the coronal plane for whole-cell patch-clamp recording of IC (external cortex layer) neurons. **(b)** Representative current-clamp traces recorded in IC neurons from wild-type and Cacna1a^S218L^ mice, demonstrating tonic firing response to increasing current injections (scale bars = 200ms, 20mV) (WT n=14; m=6, f=8; S218L n=8; m=4, f=4; no sex-related difference). **(c)** Mean input-output response data of tonic action potential frequency in response to increasing current injection over threshold. **(d)** Representative voltage-clamp traces recorded in IC neurons from wild-type and Cacna1a^S218L^ mice (scale bars = 20ms, 15pA) and mean data demonstrating no significant difference in **(e)** amplitude **(f)** or inter-event interval (frequency) of spontaneous glutamatergic synaptic activity (sEPSCs).

**
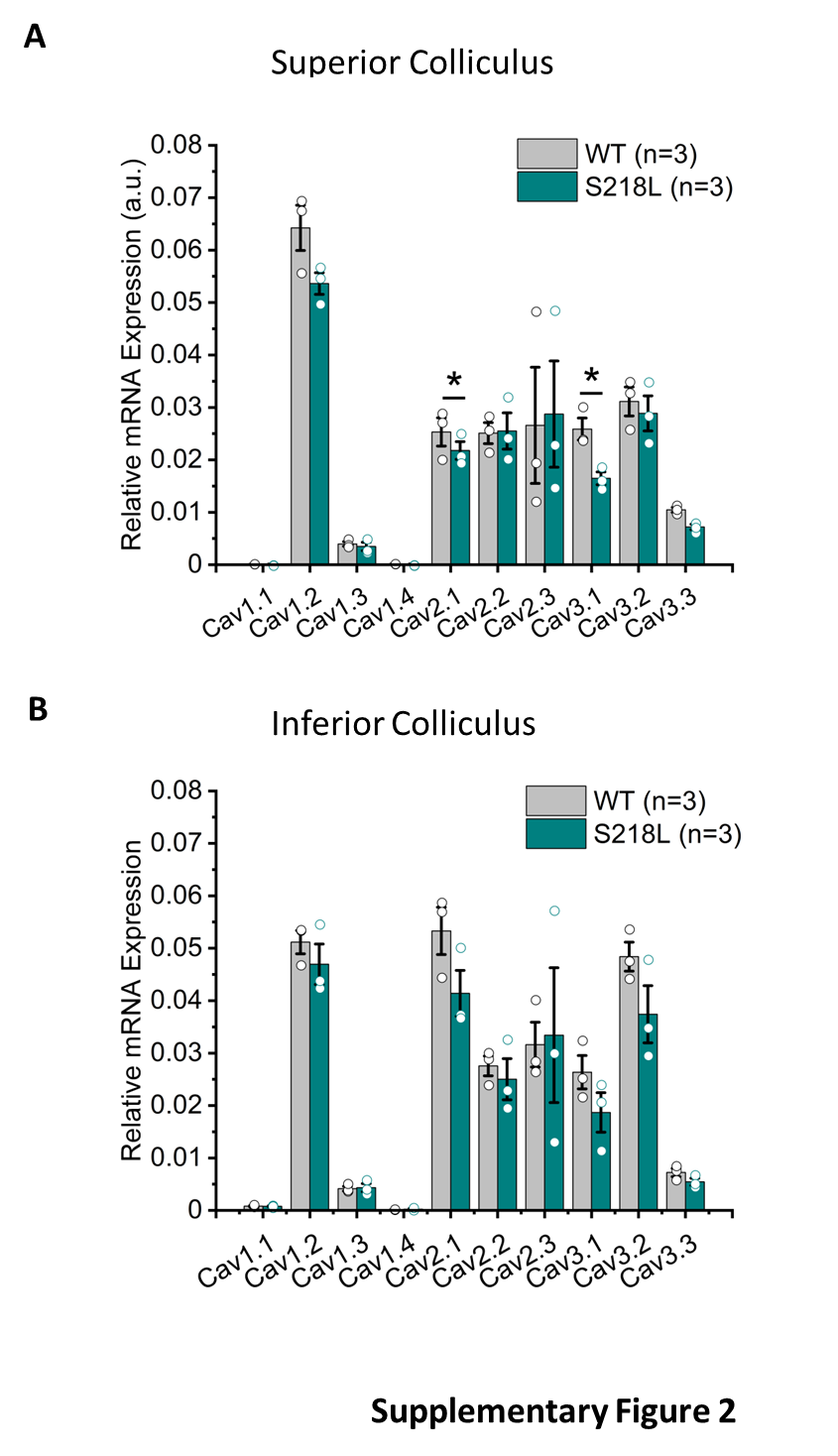
**

**Supplementary Figure 2. Ca_V_2.1 and Ca_V_3.1 T-type calcium channel mRNA levels are selectively reduced in SC of Cacna1a^S218L^ mice.** Histograms displaying mean quantitative PCR levels of calcium channel α1-subunits in samples dissected from the SC **(a)** and IC **(b)** of wild-type and Cacna1a^S218L^ mice (WT n=3; m=2, f=1; no sex-related difference; S218L n=3; m=2, f=1; no sex-related difference). Calcium channel isoform mRNA expression relative to GAPDH control mRNA. *P<0.05 Two-sample t-test.
